# An Analysis of Approaches to Reduction of HIV Stigma across the World through educational interventions: A Scoping Review

**DOI:** 10.17533/udea.iee.v42n1e06

**Published:** 2024-04-27

**Authors:** Hamideh Ebrahimi, Foroozan Atashzadeh Shoorideh, Mohammad Reza Sohrabi, Masoumeh Ebrahimi, Meimanat Hosseini

**Affiliations:** 1 Ph.D. Candidate, Student Research Committee, School of Nursing and Midwifery, Shahid Beheshti University of Medical Sciences, Tehran, Iran. Email: hamideebrahimi363@yahoo.com Shahid Beheshti University of Medical Sciences School of Nursing and Midwifery Shahid Beheshti University of Medical Sciences Tehran Iran hamideebrahimi363@yahoo.com; 2 Ph.D. Professor, Department of Psychiatric Nursing and Management, School of Nursing and Midwifery, Shahid Labbafinezhad Hospital, Shahid Beheshti University of Medical Sciences, Tehran, Iran. Email: forooata@yahoo.com Shahid Beheshti University of Medical Sciences School of Nursing and Midwifery Shahid Labbafinezhad Hospital Shahid Beheshti University of Medical Sciences Tehran Iran forooata@yahoo.com; 3 Ph.D. Professor, Department of Community Medicine, Social Determinants of Health Research Center, Shahid Labbafinezhad Hospital, Shahid Beheshti University of Medical Sciences, Tehran, Iran. Email: m_sohrabi@sbmu.ac.ir Shahid Beheshti University of Medical Sciences Social Determinants of Health Research Center Shahid Labbafinezhad Hospital Shahid Beheshti University of Medical Sciences Tehran Iran m_sohrabi@sbmu.ac.ir; 4 M.Sc. Clinical Research Development Unit of Pirooz Hospital, Guilan University of Medical Sciences, Rasht, Guilan, Iran. masome.664@gmail.com Gilan University of Medical Sciences Clinical Research Development Unit of Pirooz Hospital Guilan University of Medical Sciences Rasht Guilan Iran masome.664@gmail.com; 5 Ph.D. Associate Professor, Department of Community Health Nursing, School of Nursing and Midwifery, Shahid Beheshti University of Medical Sciences, Tehran, Iran. Email: m_hoseini@sbmu.ac.ir. Corresponding author Shahid Beheshti University of Medical Sciences Department of Community Health Nursing School of Nursing and Midwifery Shahid Beheshti University of Medical Sciences Tehran Iran m_hoseini@sbmu.ac.ir

**Keywords:** health education, social stigma, HIV, systematic review., educación en salud, VIH, estigma social, revisión sistemática., educação em saúde, HIV, estigma social, revisão sistemática.

## Abstract

**Objective.:**

To determinate the educational interventions for reducing the stigma caused by HIV worldwide.

**Methods.:**

This scoping review study analyzed all papers published from early 2000 to the end of 2022 by searching all the scientific databases, Scopus, Web of Science, PubMed, Cochrane, Embase and CINHAL. The quality assessment of the papers was done using the ROBIS tool checklist.

**Results.:**

31papers were admitted to the scoping review process. Stigma reduction intervention was founded on three parts: Society, health and therapeutic services providers, and the patients who had HIV and their families. The interventions included education on the reduction of fear, and shame, observation of protective standards, conducting tests and treatment at the above levels, as well as the support provided by the society, policymakers, religious leaders and families of patients in economic, psychological and cultural terms, together with the establishment of social centres and organization of campaigns.

**Conclusion.:**

The stigma associated with HIV is a significant obstacle before treatment, life expectancy and living quality of patients. Therefore, the stigma associated with this disease can be reduced, and the living quality of patients can be raised using approaches such as education of healthcare service providers and afflicted people, as well as economic, social, cultural, and psychological support.

## Resumen

**Objetivo.:**

Determinar las intervenciones educativas para reducir el estigma causado por el VIH en todo el mundo.

**Métodos.:**

Revisión de alcance en el que se analizaron los artículos publicados desde 2000 a 2022 recuperados en las bases de datos científicas Scopus, Web of Science, PubMed, Cochrane, Embase y CINHAL. La evaluación de la calidad de los artículos se realizó mediante la lista de comprobación de la herramienta ROBIS.

**Resultados.:**

Se admitieron 31 artículos. Las intervenciones para la reducción del estigma se basaron principalmente en tres componentes: La sociedad, los proveedores de los servicios de salud, y los pacientes con VIH y sus familias. Las intervenciones incluyeron la educación sobre la reducción del miedo y la vergüenza, la observación de las normas de protección, la realización de pruebas y el tratamiento en los niveles de atención, así como el apoyo prestado por la sociedad, los responsables políticos, los líderes religiosos y las familias de los pacientes en términos económicos, psicológicos y culturales, junto con la creación de centros sociales y la organización de campañas.

**Conclusión.:**

El estigma asociado al VIH es un obstáculo importante ante el tratamiento, la esperanza y la calidad de vida de los pacientes. Por lo tanto, es posible reducir el estigma asociado a esta enfermedad y elevar la calidad de vida de los pacientes mediante enfoques como la educación de los proveedores de servicios sanitarios y de las personas afectadas; así como el apoyo económico, social, cultural y sicológico.

## Resumo

**Objetivo.:**

Analisar as intervenções educacionais implementadas para reduzir o estigma relacionado ao HIV.

**Métodos.:**

A revisão de escopo analisou artigos publicados de 2000 a 2022 recuperados dos bancos de dados científicos Scopus, Web of Science, PubMed, Cochrane, Embase e CINHAL. A avaliação da qualidade dos artigos foi realizada usando a lista de verificação da ferramenta ROBIS.

**Resultados.:**

31 artigos foram admitidos. As intervenções para redução do estigma baseavam-se principalmente em três componentes: Sociedade, prestadores de serviços de saúde e pacientes com HIV e suas famílias. As intervenções incluíram educação sobre a redução do medo e da vergonha, adesão a normas de proteção, testagem e tratamento nos níveis de atendimento, bem como apoio fornecido pela sociedade, formuladores de políticas, líderes religiosos e familiares dos pacientes em termos econômicos, psicológicos e culturais, juntamente com a criação de centros sociais e a organização de campanhas.

**Conclusão.:**

O estigma associado ao HIV é um grande obstáculo ao tratamento, à esperança e à qualidade de vida dos pacientes. Portanto, é importante reduzir o estigma associado a esta doença e aumentar a qualidade de vida dos pacientes através de abordagens como a educação dos prestadores de cuidados de saúde e das pessoas afetadas; bem como apoio económico, social, cultural e psicológico.

## Introduction

The HIV/AIDS pandemic has been a highly significant public health challenge in the world in the past decades.^1^ In 2017, among the 36.9 million people infected with HIV worldwide, 25% were not informed about their disease, and less than 60% had taken any therapeutic measures.^2^ The global goal is to put an end to the HIV pandemic by the end of 2030; as per the United Nations’ strategy, the set goals should be achieved by 2020. These goals include the following: 90% of people living with AIDS/HIV (PLWHA) ought to get informed of their serological status; they should receive treatment, and the viral load should drop in 90% of the PLWHA who are getting treatment.^3^^,^^4^ However, various factors have acted as obstacles to achieving these goals; for instance, the stigma and discrimination associated with HIV have served as major challenges in this regard.^5^ Stigma is an undesirable or discredited characteristic attributed to a person; it also lowers the individual’s position in society’s view.^6^

Stigma is a sign of either shame or scandal; it impacts how others and the individual understand each other; besides, it questions the social identity and humanity of the individual.^7^ Stigma can destructively impact people; for instance, it can provoke family disputes and lead to social consequences and human rights violations.^8^ HIV or AIDS is a disease subject to stigma and discrimination caused by lack of knowledge, misinformation, fear of disease transmission, moral judgement about the behaviour of infected individuals and high-risk behaviours attributed to it, such as sexual contact, sex workers, and addiction. People with HIV are subject to various discriminatory behaviours such as rumours, blame, rejection, social isolation, unemployment, verbal and physical abuse, mistreatment and refusal to receive health care. ^9^^-^^11^

The stigma associated with PLWHA falls into three categories, 1- applied or experienced, 2- anticipated or perceived, and 3- internalized stigma.^12^^,^^13^ In applied or experienced stigma, the individual faces discrimination and stigma for infection with HIV; in anticipated or perceived stigma, PLWHA are expected to face stigma and discrimination from others; in internalized stigma, the infected people feel ashamed and have a negative image of themselves.^14^ The applied and anticipated stigmas cause refusal of doing tests, being afraid of disclosure of HIV status, lack of access to health and medical services and reluctance to apply for treatment;^15^^-^^17^ internal stigma causes anxiety, stress, depression, lower self-efficacy, lower self-esteem, despair and in some cases suicide. Therefore, the stigma’s impact can often be more adverse than the disease.^18^^,^^19^

To avoid stigma, PLWHA and their family members refrain from disclosing the status of their disease; it both increases the possibility of the disease spread and reduces PLWHA’s chances of seeking social support and receiving medical care.^20^ Therefore, there is a growing need to overcome the stigma associated with HIV/AIDS worldwide.^21^^,^^22^ There are different types of stigma associated with AIDS; thus, PLWHA are regularly exposed to stigma from society, health care providers, family members and themselves; thereby, most of the educational interventions done across the world have been carried out in these areas.^22^ Awareness of the interventions created for reducing stigma and applying this knowledge is essential. Therefore, the present study, aimed at determining the approaches and educational interventions for reducing the stigma associated with HIV worldwide, was done through a scoping review method.

## Methods

This research was carried out with the scoping review method based on the guidelines of the Preferred Reporting Items for Systematic Reviews and Meta-Analyses of PRISMA^23^concerning the approaches to reducing the stigma caused by HIV in the world. All the international scientific databases, Cochrane, PubMed, Web of Science, Scopus, Embase and CINHAL, were searched for the papers published from early 2000 to the end of 2022; the search was done using the Mesh keywords: Social Stigma, Stigma, Shame, HIV, Acquired Immunodeficiency Syndrome, Health Personnel, Students, Medical And other keywords includein: self-stigma, social stigma, stigma reduction, discrimination, human Immunodeficiency Virus, AIDS, intervention, people living with HIV/AIDS, PLWHA, healthcare providers, people living close to HIV, PLC. Keywords were searched in the papers’ title, abstract, and full text singularly and collectively using OR and AND operators. The search syntax in PubMed was: (((((("Social Stigma"[Mesh]) OR "Stigma"[Mesh]) OR "Shame"[Mesh]) AND "HIV"[Mesh]) OR "Acquired Immunodeficiency Syndrome"[Mesh]) AND "Health Personnel"[Mesh]) OR "Students, Medical"[Mesh].

Inclusion and Exclusion Criteria. Inclusion criteria were organized by Problem or Population, Interventions/Exposure, Comparison, and Outcome (PI(E)CO): Population: People living with HIV, health care providers, and community. Intervention/exposure: stigma reduction approaches, economic, social, psychological, therapeutic, and religious supports. Result: reduction of stigma in patients with HIV. A group of studies were excluded from this research: they were conducted as observation, review, letter to the editor, case report, and cases series, plus those without any intervention, those with low quality, or those published in a language other than English.

**Figure 1:**
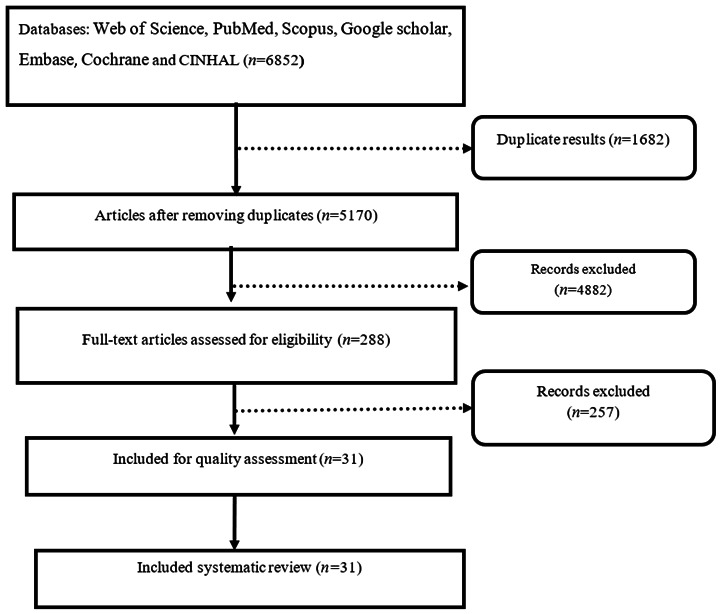
The PRISMA flow diagram

Selection of Studies. The initial search provided 6852 relevant papers. Then, the resources were entered into Endnote software to organize the studies. The duplication of resources was checked with the software, and 1682 duplicates were omitted. Then, the full text of the papers was obtained, and their titles and abstracts were evaluated, and then, 4882 articles not related to the purpose of the systematic review were omitted. Finally, the full text of 288 related articles was reviewed, and 257 studies conducted using non-interventional methods were eliminated. A total of 31 related articles were admitted to the systematic review process (Figure 1).

Quality Assessment of Papers. The quality assessment of the articles was done using the Tool to assess risk of bias in systematic reviews (ROBIS) checklist. This checklist has seven sections Random sequence generation, Allocation concealment, Blinding of participants and personnel, Blinding of outcome assessment, incomplete outcome data, Selective reporting and other sources of bias, in each section the answers Lower risk of bias, Higher risk of bias or unclear risk of bias can be selected.^24^

Data Extraction. Quality assessment and extraction of information were done independently by two researchers trained in the examination, quality assessment and evaluation of papers. Accordingly, the researchers investigated the title and abstract of the papers meeting the inclusion criteria for the systematic review. If the articles were thoroughly relevant, their full text was evaluated. If two researchers rejected an article, the reason for the rejection was mentioned. In cases of disagreement between the two researchers, the article was assessed by a third person. In order to extract data, a checklist was used; it covered the corresponding author’s profile, the place and time of the paper’s publication, the type of intervention, and the two groups of intervention and control. 

## Results

The present research investigated 31 interventional studies carried out from 2008 to 2020 on the reduction of stigma across the world. Among the conducted studies, 9 were carried out on community-based reduction of stigma, 11 were done on medical department employees and students, and 11 were interventions covering individuals with HIV. The specifications of the reviewed articles are presented in Table 1 and the results of the quality assessment of the articles are presented in Table 2.

**Table 1:** The features of the papers admitted to the systematic review

Author	Year	Country	Target group	*n*	Intervention	Effect	Quality assessment
Nyblade *et al.*^25^	2020	Ghana	Health Facility Staff	-	Education and putting up posters, banners and instructions about reducing stigma.	Reduction of Stigma	Mild
Machowska *et al.*^26^	2020	India	Health Facility Staff and Students	650	Education about stigma reduction. The education was on the epidemiology of HIV infection, the mechanisms of virus transmission, diagnostic tests, treatment options, how ART drugs work, and ethical issues related to PLWHA and their rights, the duties of HCPs, universal precautions, and measures for prevention from infection.	Increasing Knowledge and Changing Attitudes	High
Ekstrand *et al.*^27^	2020	India	Health Facility Staff	3182	Tablet-based Application. Intervention included two self-guided sessions on tablet devices and one joint skill-based session. Each tablet session included four parts and was a combination of video commentary by a narrator, interactive practices, reflection on the content and a summary of key points. Face-to-face session included experiences of PLWH around the diagnosis of HIV plus positive and negative interactions with health care system.	Reducing Misconceptions, Reducing Approval of Mandatory Policies and Discrimination and Stigma	High
Jacobi *et al.*^28^	2020	Cameroon	Secondary School Students	400	Education on the Multiple Aspects of HIV and AIDS and Stigmatization. The content of the educational sessions included 1- elaboration on the problem; 2- more understanding, less fear; 3- sex, ethics, shame and blame; 4- family and stigma; 5- coping with the stigma; and 6- moving towards action. Then the students made a story around different topics related to HIV and stigma inside society. Then, they, accompanied by a film production group, turned these scripts into plays and recorded them on DVDs.	Improving Knowledge and Performance Regarding the Reduction of Stigma	High
Camlin *et al.*^29^	2020	Kenya and Uganda	Community	144	A theoretical Model of Stigma Reduction. Reduction of internal stigma was performed through two processes of acceleration of absorption and successful use of ART; reduction of anticipated stigma was made via normalization, reduction of fear and improving the quality of HIV care.	Reduction of Stigma	High
Bauermeister *et al.*^30^	2019	Carolina	Black Men Who Have Sex with Men with and without HIV	238	Health Empowerment Intervention. web-based sessions were threefold; 1- sharing information and experiences of participants through conversations around different subject areas like getting tested, safe intercourses, dates and relationships, healthy life and living skills; 2- asking questions anonymously by participants from a doctor specializing in infectious diseases and uploading questions and answers as posts so that all participants can have access to them; 3- Sharing multimedia content including poems, videos, images and news stories and commenting on them.	Reduction of Stigma	High
Rao *et al.*^31^	2018	Chicago and Birmingham	African American Women with HIV	239	UNITY Workshop. The UNITY workshop started with a discussion around group expectations and the meaning of stigma; then, the group watched a 3-4 minutes stimulating video that was intended to provoke a discussion among the members, and following the end of the video, a facilitator led the discussion; during the discussion, the participants exchanged their reactions to the video and shared their personal experiences associated with stigma in a large group. The following practice included methods of confronting stigma, self-soothing, modelling, and practising assertiveness vs passivity or aggressiveness in response to stigma, practising self-esteem, and social support. Finally, it focused on disclosing information through case studies and role-playing.	No Effects on Stigma	Mild
Li *et al.*^32^	2018	Toronto	PLHIV* and Non-PLHIV Community Leaders	67	Collaborative Learning Approach Based on CHAMP Study. (CHAMP) evaluated the effectiveness of two group interventions, treatment, education, acceptance, commitment and social justice capacity development in reducing the stigma caused by HIV. In his study, educational activities encompassed experimental practices related to social inclusion or exclusion, critical conversations about social oppression, sharing resistance strategies, and participation in developing collective strategies to deal with HIV-related stigma and other social inequalities.	Reduction of Stigma	High
Payne-Foster *et al.*^33^	2018	Alabama	Community	12 Churches	Reducing Faith-based Stigma Using FAITH Educational Intervention. The FAITH intervention included eight modules to educate about HIV, highlight the negative impacts of stigma, encourage action to counter stigma, and support PLWH.	Reduction of Stigma	Mild
Jaworsky *et al.*^34^	2017	Toronto	Medical Students	38	Education. Education content around HIV counselling and tests was submitted to students. The student provided pre- HIV-test counselling, including obtaining sexual history and consent for taking an HIV test from the individual who had HIV. In one scenario, she delivered the negative test results and post-test counselling; in the other scenario, presented the positive test result and post-test counselling to patient.	Reduction of Stigma	Mild
Tsai *et al.*^35^	2017	Kenya	PLHIV	54	Living Intervention. A small loan about 125 US dollarswas provided for purchasing a manual water pump and related farm devices, together with an eight-session educational program on agriculture and financial management.	Reduction of Stigma	High
Prinsloo *et al.*^36^	2017	South Africa	PLHIV and Community Members	632	Stigma-reduction Community “Hub.” The intervention included 27 three-hour workshops on understanding HIV stigma for People living with HIV/AIDS (PLWHA) groups and members of the society, five educational workshops on combating HIV stigma for those interested, and weekly door-to-door education on understanding HIV stigma using pamphlets. Later, a six-session support program for both groups and eight psychotherapy group sessions were held to reduce HIV stigma in places such as churches, communities, and clinics.	Reduction of Stigma	High
Derose *et al.*^37^	2016	US	Community	-	Church-based Educational Workshop. The educational workshops were held on raising awareness about HIV, enhancing knowledge around HIV tests and HIV infection, and creating empathy for the people with HIV in churches.	Reduction of Stigma and Increase in HIV Testing	Mild
Varas-Diaz *et al.*^38^	2016	Puerto Rico	Medical Students	507	Education-based Stigma-free Spaces in Medical Scenarios. The education was provided as supplying information about HIV stigma and its consequences on service provision, the role of negative emotions like fear, shame, disgust and admiration in forming HIV stigma-based attitudes and behaviours, and skills for stigma-free interaction with PLWHA.	Reduction of Stigma	High
Pretorius *et al.*^39^	2016	South Africa	PLHIV and Close Family Members	18	Educational Workshop. The intervention consisted of a two-day workshop centred around understanding HIV stigma, identifying patients’ strengths and how they deal with responsible disclosure, and a three-day workshop on reducing HIV stigma based on the PLWH community and their families.	Reduction of Stigma from the Family	Mild
Skinta *et al.*^40^	2015	San Francisco	Gay and Bisexual Men with HIV	5	Acceptance and Compassion-Based Group Therapy. Education was held about introducing patients to the principal perspectives of the treatment group and aligning them with these perspectives. It included an introduction to stigma and identification of valuable practices as a tool to find ways to experience better life quality; The education also covered endeavours to control or avoid negative responses to HIV status and their effect on patients, value-centred behaviours and the focus on compassion, together with remembering the supportive and lovely people in one’s life. The education further discussed the acceptance, awareness and manner of alleviating concerns about HIV, self-description concerning HIV, viral load, sexual desires, experiences and history of rejection, as well as positive thoughts about oneself; other issues dealt with by the discussion were the significance of willingness to return to valuable behaviour in life, and the importance of the desire to engage in behaviours that raise fear and thoughts of HIV stigma without attempting to control or put an end to those thoughts and envisioning a caring friend.	Reduction of Stigma	Mild
Rivera *et al.*^41^	2015	New York	High-risk Population	716	An Educational Video Based on Social-cognitive Theory. The ten-minute-long educational video covered the normalization of HIV and HIV testing, education about HIV testing, promotion of HIV testing and awareness of HIV status.	Reduction of Stigma and Increase in HIV Testing	High
Pulerwitz *et al.*^42^	2015	Vietnam	Hospital Staff	795	Stigma Reduction Education. Educating the staff of the hospital covered: 1- Creation of a safe and friendly hospital environment; 2- Greater respect, care and support for the people who have HIV; 3- Developing practical skills to implement global precautionary measures systematically; 4- laying down a code of practice specifically for each hospital concerning the adoption of stigma-free practices so that the quality of services provided to HIV patients and stigma reduction approaches are enhanced.	No Effects on Stigma	High
Shah *et al.*^43^	2014	India	Nursing Students	91	Short-term Educational Program to Reduce Stigma. The intervention worked on building knowledge about the reduction of fear of the possibility of HIV transmission during short interactions, HIV epidemiology, transmission routes, misconceptions of transfer and ways of preventing HIV transmission; then, a PLWH told the story of his life before being infected with HIV; he further shared experiences of being stigmatized inside the health care environments together with some reflections on how this stigma impacted him	Reduction of Stigma	Mild
French *et al.*^44^	2014	South Africa	PLHIV and Close Family Members	83	Educational Workshop. The PLWH were educated about understanding the stigma associated with HIV, knowing personal strengths, and handling the disclosure of HIV status. Then, workshops in the form of speeches and discussions and small group activities were held in urban and rural environments with PLWH and PLC about (a) sharing information about the stigma of HIV and countering it, (b) balancing relationships between PLWH and PLC via increased interactions and contacts, and (c) empowering participants to become leaders of HIV stigma reduction using the practical knowledge and the experience provided by the programs of HIV stigma reduction and implementing them within the communities.	Reduction of Stigma	High
Barroso *et al.*^45^	2014	South Africa	HIV-Infected Women	99	Educational Video. A 45-minute video included five representations of the women infected with HIV; each woman told narratives with different topics. Narratives included the experience of an HIV-infected woman of the fear of negative social influences caused by letting others know about her HIV status, serious conflict over (not) informing her children, the importance of communication with nurses, doctors, and trusted family members and friends, the positive effects of disclosure and extra stigma and discrimination associated with being a woman, and a minority woman	Reduction of Stigma	Mild
Varas-Diaz *et al*.^46^	2013	Puerto Rico	Medical Students	507	Education-based stigma-free spaces in medical scenarios. The education was provided as supplying information about HIV stigma and its consequences on service provision, the role of negative emotions like fear, shame, disgust and admiration in forming HIV stigma-based attitudes and behaviours, and finally, skills for stigma-free	Reduction of Stigma	High
Li *et al.*^47^	2013	China	Health Staff	880	Education of popular opinion leaders (POL) about the reduction of stigma. POLs were educated about observing global precautionary standards and ensuring occupational safety, combating stigma and improving the relationship between the patient and the service provider, taking measures and attempting to take care of patients and resolve problems and create a better medical environment, and finally distributing the intervention messages to the members of their community.	Reduction of Stigma	High
Jain *et al.*^48^	2013	Thailand	Community	560	Positive Partnership Project. In the project of positive partnership, low-interest loans were granted to a pair composed of a PLHIV individual and a healthy one. They started their career by training and obtaining marketing, accounting and business management skills. Besides, stigma reduction interventions included launching HIV campaigns, providing information and education, plus holding entertainment events.	Increasing the Knowledge of HIV transmission, Reducing the Fear and Shame	High
Rao *et al.*^49^	2012	Washington	Women Living with HIV	24	Unity Workshop. Workshops included: practising 1- relaxation and self-care; 2- sharing strategies for combating other group members; 3- watching stimulating videos; 4- discussing how to handle potentially stigmatizing situations with family, at work, and in other locations; and -5 role-playing ways for handling)	Reduction of Stigma	Mild
Li *et al.*^50^	2010	China	Market Workers	4510	Effectiveness of Popular Opinion Leaders on Reduction of Stigma. POLs were taught to take on roles as the advocates of AIDS prevention in the market; afterwards, they acquired skills and knowledge on how messages about reducing HIV risk should be disseminated in everyday conversations and how they should share these skills with others.	Reduction of Stigma	High
Rimal *et al.*^51^	2008	Malawi	Community	1776	Radio Diaries Program. This radio program, aimed at reducing the stigma of HIV among the people, allowed HIV-infected men and women to talk about the events of their daily life openly.	No Effects on Stigma	Mild
Wu *et al.*^52^	2008	China	Health Staff	138	Education. Short counter-stigma education was provided around the issues appertaining to awareness of HIV policies and procedures, ensuring access to universal precautions, post-exposure prevention, enhancing knowledge of HIV transmission, and raising the comfort level of working with PLWH. Then, the program concentrated on equal medical treatment for all individuals, regardless of their social status, type of disease, or routes of infection. Afterwards, the participants discussed the possibly discriminatory language, attitudes, and behaviours commonly heard or observed in a medical environment, and then, the ways of changing them were dealt with.	Reducing negative feelings towards people with HIV	Mild
Ma *et al.*^6^	2019	World	PLWHA	23	Stigma reduction approaches among PLWHA and their families included: psycho-educational intervention, supportive intervention for adherence to treatment (antiviral treatment), psychotherapy intervention, narrative intervention and social participation intervention.	Reduce stigma	-
Thapa *et al.*^53^	2017	World	Community	21	The strategies to reduce the stigma caused by HIV and increase testing included: creating awareness, influencing normative behavior, providing support and developing regulatory laws. which could reduce the stigma caused by HIV and lead to an increase in the recruitment of people for HIV testing.	Reduce stigma	-
Feyissa *et al.*^2^	2019	World	Community	14	Information-based, skills building, structural, contact-based and biomedical interventions.	Reduce stigma	-

**Table 2:** Evaluation of the quality of the articles by level of risk of bias in included articles in the systematic review

Author	Random sequence generation	Allocation concealment	Blinding of participants and personnel	Blinding of outcome assessment	Incomplete outcome data	Selective reporting	Other sources of bias
Nyblade *et al.*^25^	High	Low	Low	Low	Low	Low	Low
Machowska *et al.*^26^	High	High	Unclear	Low	Low	Low	Unclear
Ekstrand *et al.*^27^	Low	Low	Low	Unclear	Low	Low	Low
Jacobi *et al*.^28^	High	Low	Unclear	Low	Low	Low	Unclear
Camlin *et al.*^29^	Low	Low	Low	Unclear	Low	Unclear	Unclear
Bauermeister *et al.*^30^	Low	Low	Low	Low	Low	Low	Low
Rao *et al.*^31^	Low	Low	Low	Low	Low	Low	Unclear
Li *et al.*^32^	High	Low	Low	Unclear	Low	Low	Low
Payne-Foster *et al.*^33^	Low	Low	Low	Low	Low	Low	Low
Jaworsky *et al.*^34^	High	Low	Low	Low	Low	Low	Unclear
Tsai *et al.*^35^	High	High	Low	Unclear	Low	Low	Low
Prinsloo *et al.*^36^	High	High	Low	Unclear	Low	Low	Low
Derose *et al.*^37^	High	Low	Low	Low	Low	Low	Unclear
Varas-Diaz *et al.*^38^	Low	Low	Low	Low	Low	Low	Low
Pretorius *et al.*^39^	High	High	Low	Low	Low	Low	Low
Skinta *et al.*^40^	High	High	Low	Low	Low	Unclear	Low
Rivera *et al.*^41^	High	Low	Low	Low	Low	Low	Unclear
Pulerwitz *et al.*^42^	High	Low	Low	Low	Low	Low	Unclear
Shah *et al.*^43^	High	Low	Low	Low	Low	Low	Unclear
French *et al.*^44^	High	High	Low	Low	Low	Low	Low
Barroso *et al.*^45^	Low	Low	Low	Low	Low	Low	Low
Varas-Diaz *et al.*^46^	High	Low	Low	Low	Low	Low	Low
Li *et al.*^47^	Low	Low	Low	Low	Low	Low	Low
Jain *et al.*^48^	High	High	Low	Low	Low	Unclear	Low
Rao *et al.*^49^	High	Low	Low	Low	Low	Low	Low
Li *et al.*^50^	High	High	Low	Low	Low	Low	Low
Rimal *et al.*^51^	High	High	Unclear	Unclear	Low	Low	Low
Wu *et al.*^52^	High	High	Low	Low	Low	Low	Low
Ma *et al.*^6^	High	Low	Low	Low	Low	Low	Low
Thapa *et al.*^53^	High	Low	Low	Low	Low	Low	Low
Feyissa *et al.*^2^	High	Low	Low	Low	Low	Low	Low

Overall, the findings illustrated that the stigma associated with HIV causes adverse impacts on the patients’ physical, mental and social health and acts as a significant obstacle to the prevention, control and treatment of this disease. In order to achieve control of the HIV epidemic, the reduction of stigma is emphasized across the world. The review of the studies shows that, with regard to the types of the stigma associated with HIV and the conducted evaluations, interventions have been carried out at three levels of society, healthcare service providers and patients who have HIV and their families; the following elaborates on these interventions: 

### Society-based Interventions

**Figure 2:**
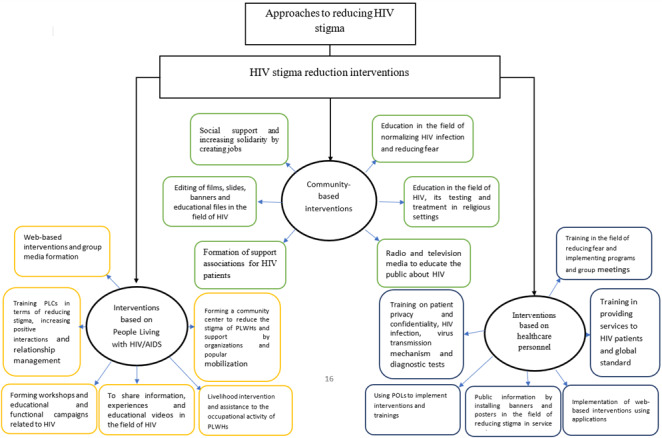
Concept map, approaches to reduce the stigma caused by HIV

In the studies centred around the society-based interventions carried out on reducing stigma in people with HIV, generally, we can point to the following component:; 1- Normalization of contracting the disease and allying fear through education and intervention programs inside the society that leads to an increase in taking HIV tests as well as applying for treatment and experiencing less isolation; 2- Opening up opportunities for social support and solidarity through the generation of jobs for the infected individuals and providing financial supports such as offering job facilities in this regard; 3- Education about HIV, it’s testing and treatment in religious environments and developing religious and educational programs for the attendance of the people suffering from HIV at these places; 4- Production of films, slides, banners and educational files about HIV for people in the community; 5- Forming associations that support HIV patients, and 6- Employing radio and television to educate the community about HIV. (Figure 2)

### Intervention on the Health and Medical Staff and Students of Medical Sciences

According to the findings, in order to maintain health, people with HIV should be provided with health care and medical services; besides, they should experience no shame, stigma and inappropriate treatment when they attend the centres. Therefore, an environment should be created where patients enjoy safety, confidentiality and empathy; the staff should also provide medical services, control the disease without fear, and behave appropriately towards patients. Accordingly, interventions were made in this regard for training and preparation of the personnel and students of medical departments. In the conducted studies, various interventions were made to reduce stigma among the people infected with HIV. Generally, the components of interventions are 1- Education about the provision of services to HIV patients; 2- Education about global standard precautions; 3- Education about preserving the privacy of patients as well as confidentiality; 4- Education about HIV infection, mechanism of virus transmission, diagnostic tests and treatment options; 5- Education about allaying fear and implementation of programs and group meetings, as well as role playings; 6- Implementation of web-based interventions with applications; 7- Informing the public by putting up banners and posters related to the reduction of the stigma in centres of service provision and public places; 8 - The utilization of POLs for conducting interventions and running educations. (Figure 2)

### PLWH-based Interventions

As per findings, the central point about reducing stigma in people with HIV is empowerment, allaying shame and fear caused by disclosure, treatment and presence in communities. Thus, these goals will be achievable via conducting intervention programs like education, preparing conditions for the presence of PLWHs in the community and their employment, and the acceptance and acceptability of these people. According to the results of the conducted studies, approaches to the reduction of stigma in PLWH included: 1- Web-based interventions and formation of mass media campaigns; 2- Sharing information and experiences and educational videos about HIV, such as programs about getting tested, safer intercourses, dating, social relationships, healthy living and living skills among PLWHs; 3- Organizing workshops and educational and functional campaigns about HIV covering positive living, easing fear and reduction of isolation, coping with stigma and applying for treatment and participation of PLWHs in these programs; 4- Living intervention and aiding PLWH’s occupational activities; 5- Forming a community centre for reduction of the stigma associated with PLWH and provision of supports by organizations, leaders and popular mobilization; 6- Educating PLCs about the reduction of stigma, increasing positive interactions and management of the relationship. (Figure 2)

## Discussion

According to the results of the present systematic review, interventions of stigma reduction working based on populations, providers of health services and PLWHs covered the elimination of the shame and concealment of the disease through normalizing the malady and allaying fear through education and intervention programs in the society; opening up opportunities for the provision of economic, social and cultural supports by the communities and their prominent members; education provided by health staff, religious leaders and notable people about HIV and taking necessary measures for the participation of the infected people in religious programs and meetings; formation of associations that support PLWH plus the employment of radio, television and web-based applications to educate the general society of PLC and PLWH; running workshops and educational and operational campaigns related to HIV; living interventions and facilitating the employment of PLWH; forming associations for the reduction of the stigma associated with PLWH; and supports provided by organizations, leaders and popular mobilization. In a review study by Andersson et al., PLWHA-based interventions included radio educational programs, provision of home-based health and medical care and family support, recruitment of affected people to income-generating activities, understanding HIV, strong interpersonal relationships, management of conflict and discrimination, setting up peer support groups, building small support teams, education, group-based behavioural interventions and social support. These factors reduced stigma and enhanced the living quality of these people.^53^ In the review study by Ma et al., which dealt with the interventions of stigma reduction in PLWHA and their families, interventions fell into five categories: 1- Psycho-educational interventions, 2- Supportive interventions for encouraging patients to comply with treatment, 3- Psychotherapy interventions; 4- Narrative interventions; and, 5- Social partnership interventions. 

The following studies covered psycho-educational interventions: education about safe behaviour, management of negative emotions, building support networks, acquiring skills of stress alleviation, reduction of stigma, calming down, anger management, decision-making skills, self-efficacy and social relationships. In terms of compliance with treatment, the goals were removing obstacles to treatment, improving access to treatment, and providing financial, nutritional, informational, and emotional support. In the area of psychotherapy, people underwent psychological and behavioural treatments. Concerning the narrative, the patients explained how they contracted the disease and the events they encountered; finally, the interventions conducted regarding social participation stressed the interaction among PLWHA, family and community. In their review, Feyissa *et al.*^2^ investigated the approaches to stigma reduction based on healthcare providers. Therefore, the components of stigma reduction fall into five categories: information-based, skills-building, structural, contact-based and biomedical interventions. These interventions were conducted as approaches providing information verbally and in writing through speeches, putting up banners and printing posters about acquiring further information on HIV and reducing stigma; a structural approach including the availability of safety and prevention equipment and related instructions, interaction strategies like activities and encouraging health workers and PLWHA to communicate; and interventions including testing, treatment and access to health care. In Mak et al. meta-analysis study, educational interventions had led to a decrease in knowledge and negative attitudes towards HIV and the caused stigma.^22^

Thapa *et al*.^55^ assessed a conceptual framework to examine the impact of interventions conducted to reduce HIV stigma on HIV testing. Therefore, the interventions were divided into four approaches: building awareness and knowledge, effect on normative behaviour, provision of support, and drafting regulatory laws. Within the awareness-building approach, effective HIV-related knowledge and norms are expanded in PWLHA and community members. In the standard behaviour approach, the attitudes and behaviours that cause stigma and the views of members of society about HIV are changed (normalized), and subsequently, more people are attracted to taking HIV tests. The approach of supporting and developing regulatory law strategies illustrates that various social and individual factors impact the mechanisms of stigma reduction; furthermore, social and legal interventions influence the implementation of the items mentioned above.^54^ In their review, Stangl *et al*.^56^ divided the factors affecting the reduction of stigma into four areas: 1- Information-based devices such as brochures, posters, and lectures; 2) Skill generation such as participatory learning sessions or role-playing to lower the rate of negative knowledge and attitudes; 3) Counselling and supporting like building support groups for PLHIV; and, 4) Communication with suffering groups like the interaction between PLHIV and the public. The review study by Mahajan et al. about the stigma caused by HIV maintains that stigma is widely used as a facilitator for the spread of the HIV epidemic; however, facilitating the provision of preventive, laboratory, and antiretroviral treatment services prevents stigma. Therefore, interventions should be carried out in the four areas of stigma and high-risk behaviour, stigma and biomedical prevention, stigma and prevention of mother-to-child transmission, and stigma and testing and treatment. 

Concerning stigma and risky behaviour, the suffering people subject to discrimination and stigma are more likely to engage in unsafe sex, risky behaviour, and concealing the disease in their sexual relationships. Thus, interventions should focus on the role of social inequalities, sexual and racial discrimination, and the reduction of risky behaviours. In the biomedical field, measures such as adult male circumcision, prevention before exposure to the virus and infection with HIV, and the use of microbicides and vaccines are the measures that restrict the spread of the epidemic. Nonetheless, the above measures, such as HIV vaccine testing and male circumcision in societies, which attach the stigma of infection to these people, lead to people’s unwillingness to comply with these principles. Regarding the prevention of the mother-to-child transmission of disease, pregnant women do not want to be tested and disclose their disease status as they are afraid of the stigma, discrimination and violence from their husbands and families. Therefore, it is necessary to educate the community, pregnant women and women of reproductive age about the services provided to prevent the mother-to-fetus transmission of the disease so that the acceptability of services is improved and the stigma is reduced. According to the treatment and testing approach, an obstacle to taking an HIV test and its treatment is stigma. But on the other hand, the lack of proper access to antiretroviral treatment in countries with limited resources and the lack of facilitation of testing act as the actual driver of not taking tests and applying for treatment. For instance, people with advanced HIV/AIDS who have clinical symptoms can no longer work; they also experience serious stigma. Therefore, treatment of these people and reduction of clinical symptoms allow these people to return to a productive social and financial life. Overall, the facilitation of HIV testing and treatment can turn this disease into a curable, chronic disease; the stigma caused by it can also be reduce. In addition, it is widely acknowledged that education plays a crucial role in mitigating the stigma surrounding HIV globally. Therefore, various approaches should be employed to present the message in different contexts. The target groups can also affect these conditions; that is, the type of training can differ depending on the illness and circumstances at that particular time. As a result, virtual education should be given more importance during the COVID-19 pandemic and effective methods should be used to implement it, such as developing an easily navigable platform for the general public. Previous research has shown that conditions prior to the pandemic were different. because of the numerous impacts COVID-19 has had on practically every organ in the body, including the liver, kidneys, lungs, eyes, and so forth. Virtual classes are important because, quite naturally, people's fear of getting this virus can make them avoid attending training sessions in person.^57^^-^^65^

In general, the conducted studies have introduced various approaches to reducing the stigma caused by HIV; they also evaluated these approaches as interventions in different communities. Having an objective assessment of the condition and performance of AIDS patients and the attitude of nurses in relation to the insight, customs and norms of patients with AIDS is effective in making the right decisions in interacting with patients. Due to limitations in time, resources and performance, nurses need to benefit from the best evidence-based resources to improve their skills, and the results of review studies are very effective to improve knowledge and implement appropriate approaches to reduce stigma. The present research elaborated on these interventions and summarized them. However, the stigma cannot be controlled only by intervention in a specific group or population; diverse factors influence it in communities, families, suffering people and the policies related to prevention, diagnosis and treatment facilities. Besides, complex relationships exist among them, and as noted earlier, these factors have mutual effects in many cases. People with HIV will be deprived of a normal life, high-quality living, life expectancy and treatment enjoyed by all people suffering from other chronic diseases. The prevention and control of this epidemic will not be possible unless the interventions described above are implemented as a general policy in societies.

Limitations. Limitations of the study include 1- Not using the same tool to check the reduction of stigma in studies; 2- Examining populations with different economic and social conditions; 3- Selection of diverse control groups in studies; and 4- Conduction of studies in different times, and the possibility of the effect of therapeutic, cultural and social developments on the studies.

Conclusion. The stigma associated with HIV is a significant obstacle to treatment, life expectancy and living quality of patients. Therefore, the education of society, healthcare service providers, and afflicted people can reduce the stigma caused by this disease and raise the living quality of patients. Furthermore, economic, social, cultural, and psychological support can reduce the stigma of this disease and increase the living quality of the patients. Thus, it is recommended to conduct educational interventions within laws, instructions and programs in communities, health and medical and educational centres so that people suffering from this disease can enjoy a quality life.
